# Enzymatic and Cellular Degradation of Carbon-Based Biconcave Nanodisks

**DOI:** 10.3390/mi13071144

**Published:** 2022-07-19

**Authors:** Zhiyong Wei, Qingxin Mu, Hui Wang, Guanyou Lin, Miqin Zhang

**Affiliations:** 1 Department of Materials Science and Engineering, University of Washington, Seattle, WA 98195, USA; zywei@dlut.edu.cn (Z.W.); qmu@uw.edu (Q.M.); wanghui3607@gmail.com (H.W.); linguany@uw.edu (G.L.); 2 State Key Laboratory of Fine Chemicals, Department of Polymer Science and Engineering, School of Chemical Engineering, Dalian University of Technology, Dalian 116024, China

**Keywords:** carbon-based nanoparticles, enzymatic degradation, cellular degradation, biconcave nanodisks

## Abstract

The assessment of the biodegradability of nanomaterials is of pragmatic importance for understanding the interactions between nanomaterials and biological systems and for the determination of ultimate fate of these materials as well as their potential use. We recently developed carbon-based biconcave nanodisks (CBBNs) serving as a versatile nanocarrier for enhanced accumulation in tumors and combined photothermal-chemotherapy. Here, we investigate both the enzymatic and cellular degradation of CBBNs by monitoring their cellular response with electron microscopy, near-infrared absorbance spectroscopy, and cell viability and oxidative stress assessments. Our results show that CBBNs underwent significant degradation in solutions catalyzed by horseradish peroxidase (HRP) and hydrogen peroxide (H_2_O_2_), or in the presence of macrophage cells. The ability of CBBNs to be degraded in biological systems provides suitability for their future biomedical applications.

## 1. Introduction

Over the past few decades, the rapid development of carbon nanomaterials, including carbon nanotubes, graphene, and their derivatives, has attracted enormous attention, because of the unique electronic, optical, thermal, and mechanical properties these nanomaterials provide [1,2,3]. Extensive efforts have been devoted to exploit the application of these materials in medicine, including diagnostics, therapeutics, and prevention [4,5,6,7]. The successful applications of these carbon-based nanomaterials in medicine depends ultimately on how they interact with biological systems, which in turn determines their functions and fates [8,9].

Consequently, the evaluation of biodegradation of carbon nanomaterials is of pragmatic importance for applications of these materials in medicine; it helps to understand the long-term behavior of these nanomaterials including accumulation, degradation, and/or excretion in tissues [10]. Studies have shown that carbon nanomaterials can be degraded enzymatically by peroxidases in plants, animals, and humans, such as horseradish peroxidase (HRP) and myeloperoxidase (MPO) under the presence of a low concentration of hydrogen peroxide (H_2_O_2_) [11,12,13,14,15]. Degradation measurements have been commonly carried out in solutions on conventional carbon nanomaterials and their surface modification derivatives for drug delivery purposes [16,17,18,19]. Nevertheless, the degradation in solutions doesn’t necessarily indicate that those nanomaterials would be also degradable in a cellular environment, where they would locate when nanoparticles are used as drug carriers. Several studies have investigated the cellular interactions of the materials such as cellular degradation of carbon nanotubes and nanohorns in recent years [20,21,22,23,24,25]. The cellular degradation is closely related to the fate of carbon nanomaterials in human body. Therefore, it is critical to understand whether the degradation would occur in a physiologically relevant environment such as macrophage cells, especially for novel carbon nanomaterials.

Carbon-based biconcave nanodisks (CBBNs) have received much attention in development of multifunctional nanocarriers [26,27,28]. Investigations have demonstrated CBBNs’ superior biological properties over their spherical counterparts, including cellular internalization, biodistribution, circulation, accumulation, and penetration [29,30]. We have recently developed novel CBBNs and demonstrated their enhanced tumor accumulation and combined photothermal-chemotherapy [31].

In this study, we investigated both enzymatic degradation using a horseradish peroxidase (HRP)-catalysed reaction in solution as well as the cellular degradation behaviors of CBBNs with macrophages. This study can support further understanding of the biological interaction and fate of these CBBNs.

## 2. Materials and Methods

### 2.1. Materials

CBBNs were synthesized by a hard templating and hydrothermal etching method and characterized as described previously [31]. Briefly, monodispersed Fe_3_O_4_/carbon core/shell template nanoparticles were first prepared by a one-pot solvothermal method using ferrocene as the single precursor. Next, the magnetic core was dissolved using an acid solution, leading to the formation of CBBNs. The phase composition of the CBBNs consists of π carbon only, which has been confirmed by X-ray powder diffraction patterns and Raman spectroscopy [31]. Considering the existence of hydrophilic functional groups, including hydroxyl and carbonyl, the CBBNs also contained small fraction of oxygen besides the carbon component. Horseradish peroxidase (HRP) type VI (>250 units/mg), dry ethanol, and 30% hydrogen peroxide (H_2_O_2_) were purchased from Sigma Aldrich. 

### 2.2. Enzymatic Degradation of CBBNs in the Presence of HRP and H_2_O_2_

A suspension of CBBNs (30 μg/mL) in 5 mL of phosphate-buffered saline (PBS) was sonicated in a water bath for 1 min, followed by addition of HRP to maintain the final 25 units/mL enzyme activity in the solution. To enable the enzymatic activity, 10 μL of H_2_O_2_ (800 μM) was added to the solution every day. The suspension was stirred in water bath at 37 °C. Aliquots (500 μL) were taken at days 0, 5, 10, 20, and 25 and stored at –20 °C until characterization by TEM and near-IR absorption spectroscopy.

### 2.3. Transmission Electron Microscopy (TEM)

TEM samples were prepared by centrifuging 250 μL of CBBN suspensions at 6000 rpm for 5 min. After removal of the supernatant, the sample was washed three times with ethanol and diluted in deionized water, followed by centrifugation. Finally, CBBNs were resuspended in 1 mL of ethanol under sonication, and 10 μL of the suspension was then dropped on a lacey carbon grid and dried in ambient conditions overnight for TEM imaging (FEI, Hillsboro, OR, USA, 200 keV).

### 2.4. Near-Infrared (IR) Absorption Spectroscopy

Near IR absorption spectra were acquired on a UV-Vis-NIR spectrometer (Agilent Technologies, Santa Clara, CA, USA). The samples were diluted 10× with ethanol. 

### 2.5. Cellular Degradation of CBBNs in Macrophages

Mouse macrophage cells (RAW264.7, ATCC) were used in this degradation study. Cells were grown in RPMI-1640 media supplemented with 10% FBS and 1× antibiotics. Cells were maintained in a CO_2_ incubator with 5% CO_2_ and 95% humidity.

Cells were seeded in a 6-well plate (1 × 10^5^ per well) and incubated overnight. Cell culture media were then removed and replaced with fresh media containing CBBNs with final concentration of 3 and 6 μg/mL. The CBBNs were incubated with cells for 24 h. Each condition was triplicated (*n* = 3). The CBBN-containing medium was then removed and replaced with fresh medium. Cells were then allowed to grow for 2, 4, and 8 d under normal condition. Cells were passaged and split into 10-cm dishes when cells achieved ~90% confluency. At each time point, the cell culture medium containing dead cells and all carbon materials was collected in a 15-mL conical tube. Cells were trypsinized, aspirated and mixed with the original medium and centrifugated at 500× *g* for 3 min. Cell pellets were then lysed by cell lysis buffer. CBBN content was determined by near-IR absorbance [32]. A standard curve was established with absorbance values of pure CBBN suspensions in ethanol at 970 nm. Cell suspension was diluted with ethanol and the absorbance of the diluted cell suspension at 970 nm was used to calculate the CBBN amount.

### 2.6. Cellular Reactive Oxygen Species (ROS) Detection

In another experiment, cellular oxidation of dihydroethidium was used to monitor the generation of ROS followed by CBBN incubation. RAW264.7 cells were seeded in 6-well plates (1 × 10^5^/well) and incubated overnight. CBBNs were added into cell culture medium with final concentration of 3 and 6 μg/mL, and incubated with cells for 2, 4 and 8 days. After that, cells were incubated with dihydroethidium (5 mM in cell culture medium) for 30 min. Cells were then trypsinized and resuspended in cold PBS and analyzed by flow cytometry (FACSCanto II, BD Biosciences) (Ex: 488 nm, Em: 585 nm, bandwidth: 42 nm). Each condition was triplicated (*n* = 3).

### 2.7. Cell Viability

RAW264.7 cells were seeded in 96-well plates (5 × 10^3^/well) for the viability assay. Cells were incubated with CBBNs (3 or 6 µg/mL) for 24 h and the medium was then replaced with fresh medium containing no CBBNs. Cells were then further cultured for 2, 4, and 8 days before the Alamar Blue assay. Specifically, the medium was replaced with cell culture medium containing the Alamar Blue reagent and incubated for 2 h. Following the incubation, a microplate reader (SpectraMax i3 multimode microplate reader, Molecular Devices) was used to determine the fluorescence intensity of the dye (550ex/590em nm). The fluorescence intensity from CBBN treated cells was compared to those from untreated control cells to determine percent viability. Each condition was triplicated (*n* = 3).

## 3. Results

**Figure 1:**
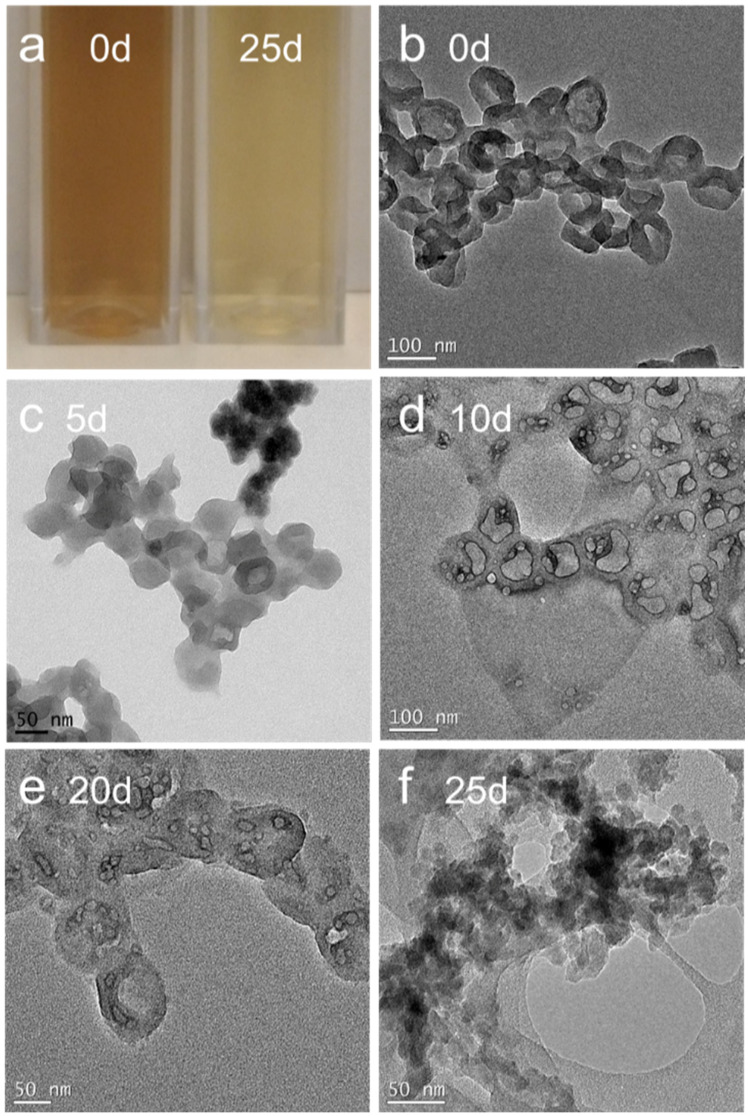
Enzymatic degradation of CBBNs incubated with HRP and H_2_O_2_. (**a**) Photograph of CBBNs dispersions before and 25 days of the incubation. (**b**–**f**) TEM images of CBBNs, acquired at various incubation times. Scale bars represent 50 or 100 nm as specified.

Previous studies have shown that carbon nanotubes can be enzymatically degraded by peroxidase catalysis with the assistance of H_2_O_2_ [11,12,13,14,15]. A similar degradation behavior was expected for CBBNs due to their similar π carbon framework characterized previously by X-ray powder diffraction and Raman spectroscopy [31]. To confirm this, we conducted experiments of enzymatic degradation on CBBNs under similar conditions. CBBNs were added into solution containing HRP (25 U/mL) and H_2_O_2_ (800 μM) and incubated at 37 °C for up to 25 days. Figure 1a shows a photograph of a vial containing CBBNs prior to (left) and 25 days after (right) incubation. The solution transformed to a lighter color after the incubation, conforming the degradation of CBBNs. To further verify the enzymatic degradation of CBBNs, TEM was used to examine the morphological changes of CBBNs (Figure 1b–f). The TEM images were acquired before sample incubation and then every five days after incubation. As shown, before sample incubation (Figure 1b), CBBNs displayed a bowl-shape morphology with a “radius” of ~50 nm. At day 5, the outer rims of CBBNs appeared blurred in the image, and the morphology is no longer bowl-shaped. At day 10, most of CBBNs completely collapsed into carbon sheets. At day 20, the carbon sheets further broke down into much smaller sheets. At day 25, individual particles aggregated and were identifiable, and appeared similar to amorphous carbon or graphite-like carbon nanoparticles reported elsewhere [20]. This result suggests that CBBNs underwent a substantial degradation when incubated with HRP and H_2_O_2_. 

**Figure 2:**
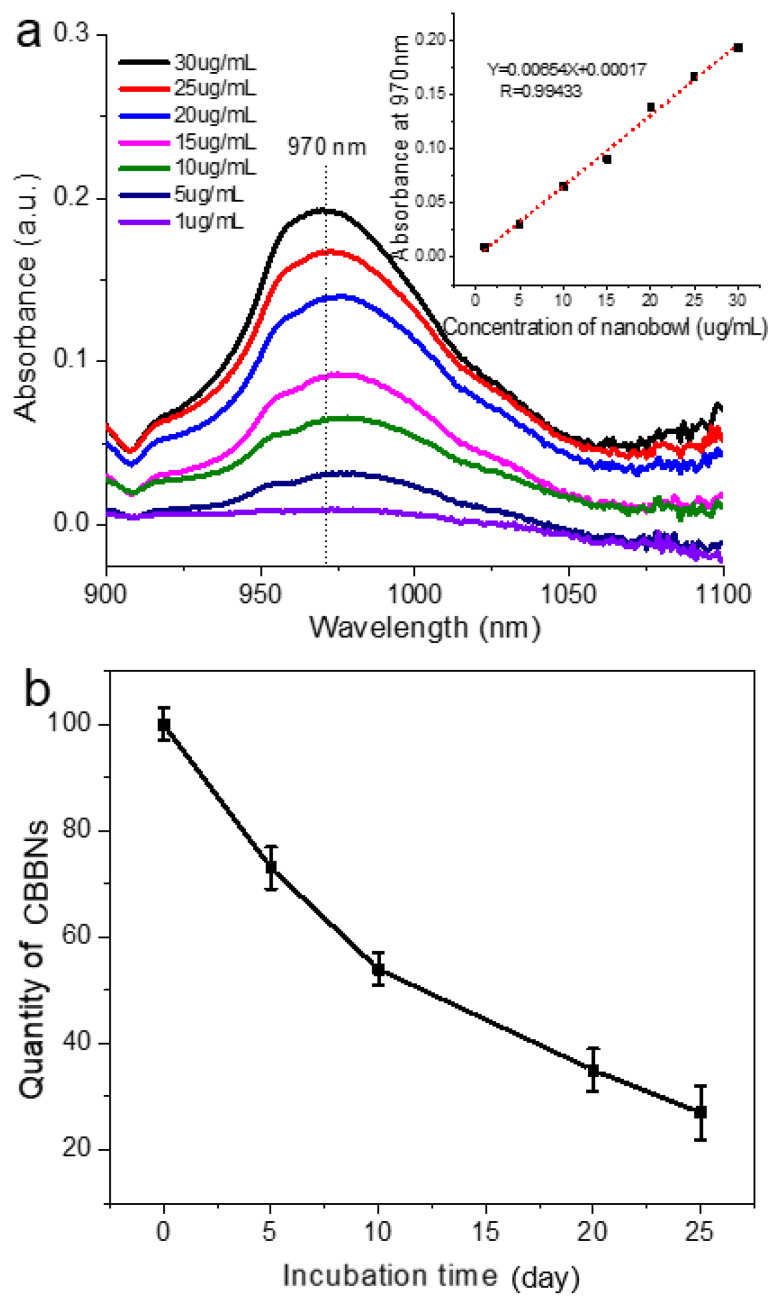
Degradation of CBBNs. (**a**) Near-IR absorption spectra of CBBNs dispersed in ethanol solution after sonication for 20–30 min. Inset: The standard calibration that relates the amount of CBBNs to the absorbance at 970 nm. (**b**) Quantitative analysis of enzymatic degradation of CBBNs incubated with HRP and H_2_O_2_ as indicated by percentage of absorbance at 970 nm.

The amount of CBBNs was also quantified by absorbance spectroscopy at various time points over the 25-day incubation time. To do this, a standard near-IR absorbance curve was first established to relate the mount of CBBNs in suspension with absorbance at wavelength of 970 nm as follows. The spectra were acquired from sample solutions containing different CBBN concentrations dispersed in ethanol. Figure 2a shows the near-IR absorption spectra acquired from these solutions with wavelengths from 900 to 1100 nm. This wavelength range was chosen to avoid spectral interference from the solvent (ethanol) and water molecules. The characteristic peak at 970 nm, which is close to the broad S2 semiconducting band of the carbon nanotubes between 1000 and 1100 nm [11], strongly depends on the concentration of CBBNs. Consequently, the calibration curve of CBBNs for the absorbance as a function of the concentration of CBBNs was established at 970 nm (insert of Figure 2a). The quantity of CBBNs sampled at various incubation times was then evaluated based on this standard curve. Figure 2b shows that the quantity of CBBNs continually decreased overtime during enzymatic degradation. After 25 days of incubation, only ~30% of the initial quantity of CBBNs remained. Insets are TEM images showing morphological changes of CBBNs over time.

**Figure 3:**
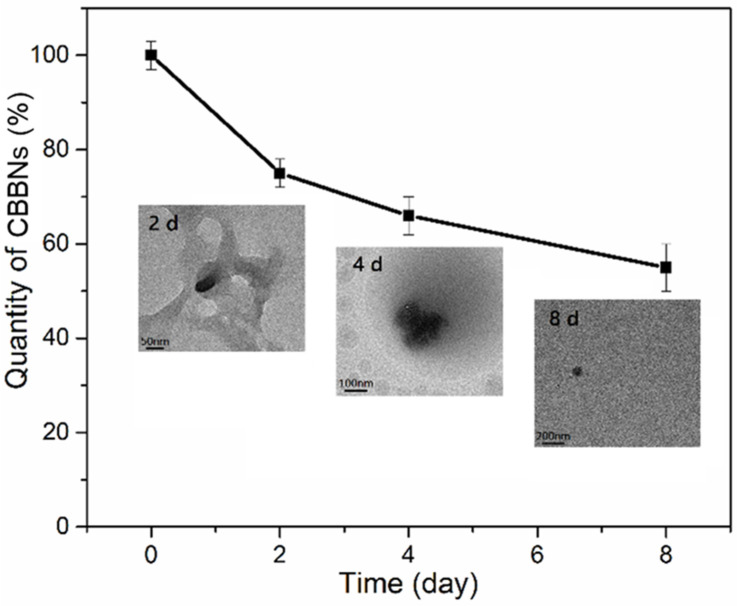
Quantitative analysis of cellular degradation of CBBNs in RAW 264.7 macrophages. Insets are TEM images showing morphological changes of CBBNs during cellular degradation. Scale bars represent 50 nm at 2 d, 100 nm at 4 d, 200 nm at 8 d.

To investigate the cellular degradation behaviour of CBBNs, CBBNs were incubated with mouse macrophage cells (RAW 264.7) at a concentration of 6 μg/mL for up to eight days. The cells were lysed at pre-set incubation time points, and the quantity and morphology of CBBNs in cell lysates were determined by absorbance and TEM, respectively. Near-IR absorption measurements indicated that the quantity of the CBBNs in cells was approximately 60% of its initial value after eight days of incubation (Figure 3). The TEM images (Figure 3, insets) showed morphological changes of CBBNs (black dots in images) and the size of CBBNs decreased gradually over time. Furthermore, the bowl-like morphology of CBBNs was no longer present after two days of incubation.

**Figure 4:**
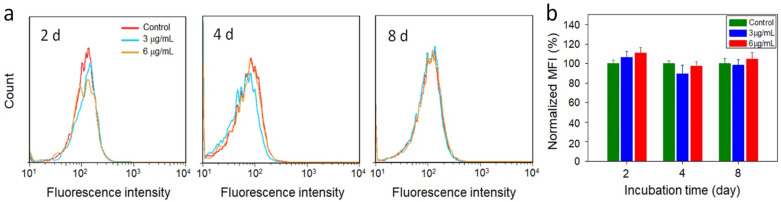
Evaluation of cellular degradation of CBBNs. (**a**) ROS production in RAW264.7 cells by flow cytometry. Cells were incubated with CBBNs at 3 or 6 g/mL for 24 and then for 2 (**left**), 4 (**middle**) and 8 (**right**) days. (**b**) Normalized mean fluorescence intensity (MFI) of cells from flow cytometry plots in (**a**).

**Figure 5:**
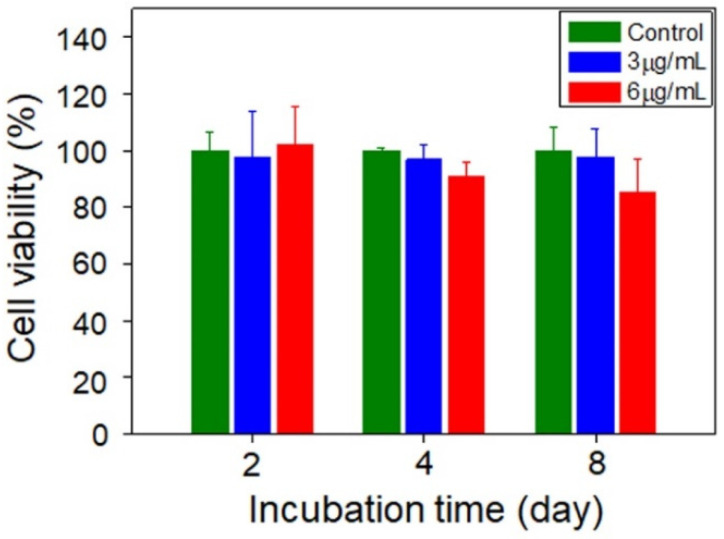
Viability of RAW264.7 cells incubated with different concentration of CBBNs as a function of incubation time.

It was reported that after the uptake of carbon nanoparticles by RAW 264.7 macrophages, reactive oxygen species (ROS) were generated due to oxidative stress [33]. ROS play an important role in cellular oxidative stress induced by nanomaterials. ROS are also known to be an oxidizing agent, which could in turn promote enzymatic degradation of nanomaterials [15]. Thus, the cellular oxidation of dihydroethidium, a marker that can be oxidized by superoxide to form fluorescent 2-hydroxyethidium, measurable by flow cytometry, was evaluated to monitor the generation of ROS. Followed by CBBN incubation with RAW 264.7 cells, the marker was added to cells and cellular fluorescence was detected by a flow cytometer. As shown in Figure 4, the ROS levels in the cells incubated with CBBNs (3 or 6 μg ml^−1^) were slightly higher than control cells receiving no CBBN treatment at day 2 after treatment but returned to the normal value at day 8 (Figure 4a). This indicates a moderate and transient effect of CBBNs on cellular oxidative stress. A slightly elevated ROS level could contribute to degradation of the CBBNs due to the possible oxidation of CBBNs by ROS. During degradation of CBBNs in cells, fewer CBBNs remained to induce further production of ROS, therefore resulting in the reduction of ROS over time. As oxidative stress is a major cause of reduced cell viability in nanomaterials, the viability of RAW 264.7 cells was tested with the Alamar Blue assay under the same conditions (incubation time and CBBN concentrations) as the evaluation of ROS. Figure 5 shows that slightly lower viability observed (~80%) at higher CBBNs concentration (6 μg mL^−1^) after cells were treated for long time (8 days) suggests that CBBNs did not cause appreciable cytotoxicity. As we did not observe any cell death or morphological changes under microscope, we speculate that the ~20% inhibition might be contributed from slight inhibition on cell proliferation. The cytotoxicity mechanisms at high doses require further in-depth investigations. These experiments indicate that CBBNs are not only biodegradable, but also biocompatible to macrophage cells.

In summary, we demonstrated the biodegradation behaviours of CBBNs by HRP catalysed oxidation and the presence of mouse macrophages. With TEM images, we showed that the bowl-like structure of CBBNs was destroyed gradually during the enzymatic degradation process. The similar structure disruption was also found when CBBNs were placed in a suspension of cellular lysates, and this was attributed to cellular degradation. Near-IR absorption quantitative analysis revealed that the quantities of the CBBNs were approximately 30% and 60%, respectively, of the original values after 25 days and two days of incubation with macrophage cells, respectively. Degradation of these CBBNs caused moderate generation of ROS, which slightly reduced cell viability only under a high concentration of CBBNs and after a long incubation time. Our experiments show that CBBNs have good biocompatibility and can be degraded by either enzyme or macrophage cells. These properties are favourable for in vivo biomedical applications, such as drug delivery and photothermal therapy.

## Data Availability

Not applicable.
